# Extracellular matrix surface regulates self-assembly of three-dimensional placental trophoblast spheroids

**DOI:** 10.1371/journal.pone.0199632

**Published:** 2018-06-25

**Authors:** Michael K. Wong, Sarah A. Shawky, Aditya Aryasomayajula, Madeline A. Green, Tom Ewart, P. Ravi Selvaganapathy, Sandeep Raha

**Affiliations:** 1 Graduate Program in Medical Science, McMaster University, Hamilton, Ontario, Canada; 2 Department of Pediatrics, McMaster Medical Centre, Hamilton, Ontario, Canada; 3 Department of Mechanical Engineering, McMaster University, Hamilton, Ontario, Canada; 4 Evik Diagnostics, Ottawa, Ontario, Canada; Technische Universitat Dresden, GERMANY

## Abstract

The incorporation of the extracellular matrix (ECM) is essential for generating *in vitro* models that truly represent the microarchitecture found in human tissues. However, the cell-cell and cell-ECM interactions *in vitro* remains poorly understood in placental trophoblast biology. We investigated the effects of varying the surface properties (surface thickness and stiffness) of two ECMs, collagen I and Matrigel, on placental trophoblast cell morphology, viability, proliferation, and expression of markers involved in differentiation/syncytial fusion. Most notably, thicker Matrigel surfaces were found to induce the self-assembly of trophoblast cells into 3D spheroids that exhibited thickness-dependent changes in viability, proliferation, syncytial fusion, and gene expression profiles compared to two-dimensional cultures. Changes in F-actin organization, cell spread morphologies, and integrin and matrix metalloproteinase gene expression profiles, further reveal that the response to surface thickness may be mediated in part through cellular stiffness-sensing mechanisms. Our derivation of self-assembling trophoblast spheroid cultures through regulation of ECM surface alone contributes to a deeper understanding of cell-ECM interactions, and may be important for the advancement of *in vitro* platforms for research or diagnostics.

## Introduction

The human placenta is pivotal in the growth and survival of the fetus during pregnancy due to its involvement in maternal-fetal exchange, immune and barrier protection, and endocrine regulation [1, 2]. To achieve an understanding of the complex processes underpinning this rapidly developing tissue requires a diverse range of experimental approaches including both *in vivo* and *in vitro* models. There has recently been great interest in emulating placental barrier function utilizing *in vitro* models comprised of monolayers of trophoblast cells or more complex assemblies of multiple cell types referred to as microphysiological systems [3–5]. However, many of these *in vitro* platforms are developed in the absence of the non-cellular scaffold present *in vivo* known as the extracellular matrix (ECM) [6, 7]. The ECM is not routinely incorporated in most culture systems, where cells are simply cultured on two-dimensional (2D) polystyrene or glass surfaces. The physical properties of these 2D surfaces are known to be quite distinct from that which exists *in vivo* [8]. Considering that the ECM provides numerous biochemical and biomechanical cues that are important for regulating cell behavior [9], the incorporation of ECM for *in vitro* modeling and testing may be of central importance to accurately understanding placental barrier function.

While the importance of considering the three-dimensional (3D) ECM for *in vitro* cell culture models is becoming evident [10], our understanding of the regulatory role of cell-matrix interactions on cell function is still incomplete. Specific to placental development, trophoblast cells grown on various ECMs have demonstrated phenotypic changes, such as altered gene and protein expressions, that are indicative of a more differentiated population [11–15]. Yet, the functional consequences of these biointerface-driven changes in phenotype have yet to be fully elucidated. In particular, there is a disparity in our understanding of parameters such as surface thickness and stiffness in the context of trophoblast biology. As ECM properties may provide key cues to direct cell fate and behaviour [16, 17], inconsistencies in tuning the growth surface may have implications on the translatability of resultant findings. Hence, there is a need to understand how the ECM parameters employed during *in vitro* culture impact trophoblast growth and function. While the literature does not provide highly defined measures of human placental ECM thickness and stiffness, we do know that changes in these parameters are associated with placental pathologies such as intrauterine growth restriction [18]. Therefore, when developing *in vitro* microphysiological systems, failure to clearly define the ECM may result in abnormal representation of cellular function.

In the current study, we investigated the impact of the ECM on placental trophoblast cells *in vitro*. We hypothesized that altering ECM surface thickness and stiffness would affect cellular organization, function, and expression profiles. A deeper understanding of the biointerface-driven effects of ECM thickness on trophoblast cell phenotype will be fundamental in the development of more *in vivo*-like models for pregnancy research, drug/toxin testing, and prognosis of placental pathologies.

## Materials and methods

### ECM hydrogel surface fabrication

Collagen I (Corning; 2 mg/mL) and Matrigel (Corning; 5 mg/mL) were used as ECM hydrogels for this study as they are two of the most commonly-utilized ECM growth surfaces for cells [19, 20]. Matrigel is a reconstituted basement membrane extract from the Engelbreth-Holm-Swarm mouse sarcoma, which consists of approximately 60% laminin, 30% collagen IV, 8% entactin, and other proteins and growth factors (Corning). Growth factor-reduced Matrigel was used in this study to minimize the effect of growth factors and to increase comparability to collagen I, which is also growth factor-free [21]. Two surface thicknesses (50 and 250 μm) were selected on basis of the most commonly used ranges previously seen in literature for trophoblast culture [11–15]. In order to calculate the volume of hydrogel required to produce a specific surface thickness, the following equation was used: *Volume = (surface area of cell growth) x (calculated thickness)*. A controlled volume of liquid hydrogel material was deposited onto glass or polystyrene surfaces via micropipetting and spread evenly over the surface. Matrigel was gelled via incubation at 37°C for 1 hour. Collagen I was gelled via the addition of 10X phosphate-buffered saline (PBS) and 1N sodium hydroxide, and incubated at 37°C for 1 hour, according to the manufacturer’s protocol.

### Analysis of ECM surface properties and mechanical testing of substrate stiffness

Analysis and mechanical testing of ECM surfaces were carried out using the MicroSquisher instrument (CellScale). Images were captured using the MicroSquisher camera and the data was recorded using the SquisherJoy software (CellScale). Actual thicknesses of the surfaces were measured by cross-sectional imaging of the hydrogel and glass coverslip and using a measurement tool within the SquisherJoy software. A total of five measurements were taken per sample along the edge to the centre of the ECM surface.

A 2 mm x 2 mm steel plate glued to a cylindrical cantilever of 203.2 μm diameter was used to perform the mechanical testing for the experiment. All samples were tested in phosphate-buffered saline bath at room temperature. The cantilever was lowered until it made gentle contact with the top of the ECM sample. Samples were compressed to 10% engineering strain at a strain rate of 1.61 μm/second and held at a constant deformation for 10 s followed by a release strain rate of 1.61 μm/second. Force was measured during the compression, deformation and release cycle. All the gels showed an elastic region between 3–5% of the strain values which were used for analysis. Substrate stiffness was determined by assessing the force required to compress the sample to a constant displacement.

### Cell culture

BeWo cells (ATCC) are one of the most extensively-used cell lines in placental trophoblast research to model villous trophoblasts, syncytial fusion, and many aspects of placental function and disease [22, 23]. BeWo cells were cultured at 37°C in 95% room air/5% CO_2_ in F-12 media (Corning) supplemented with 10% fetal bovine serum, 1% L-glutamine, and 1% penicillin-streptomycin. The media was changed every two days. Cells between the passages of 10–15 were used for all experiments, and seeded at an initial density of 1x10^4^ cells/cm^2^ on glass coverslips or 6-well polystyrene plates that were either uncoated (2D control) or coated with varying thicknesses of collagen I or Matrigel.

### Live cell imaging of cellular organization

Cells were imaged under a phase-contrast filter and images captured using an AE2000 inverted microscope (Motic) and Moticam X2 camera (Motic). Images of cellular organization were captured at 4x objective magnification.

### Immunofluorescence

Cells were fixed for 10 minutes in 2% paraformaldehyde with 0.1% glutaraldehyde and permeabilized for 5 minutes with 0.1% Triton X-100 in PBS. Samples were then blocked for 2 hours using 0.01% Tween-20, 10% goat serum and 1% bovine serum albumin (BSA) in PBS. Afterwards, samples were incubated with either Anti-E-Cadherin primary antibody (Abcam; ab40772; rabbit monoclonal; 1:500) overnight at 4 degrees and then incubated with Goat Anti-Rabbit IgG H&L Alexa Fluor® 488 secondary antibody (Abcam; ab150077; goat polyclonal; 2μg/mL) for 1 hour, or with CytoPainter Phalloidin-iFluor 555 reagent (Abcam; ab176756; 1:1000) for 1 hour. All blocking and incubations were performed at room temperature, unless otherwise stated. Samples were counterstained with 4′,6-diamidino-2-phenylindole dihydrochloride (DAPI; Santa Cruz; 1.5 μg/mL) and mounted onto glass slides using Fluoromount™ Aqueous Mounting Medium (Sigma-Aldrich). Slides were visualized using an Eclipse Ti-E Inverted Fluorescence Microscope (Nikon). Z-stack images were taken in 0.5 μm steps to capture all layers. Images were analyzed using Fiji (National Institutes of Health) and NIS Elements software (Nikon). To assess syncytial fusion, E-Cadherin was visualized to identify cell borders (as E-Cadherin is localized to the plasma membrane [24]) and DAPI to identify cell nuclei. When merged, E-Cadherin and DAPI enabled the visualization of syncytial fusion [25]. Cell fusion may be quantified with the following equation [26]: *Total Fusion Percentage (%) = (Number of nuclei in syncytia/Total number of nuclei) x 100%*. Cell spread area was determined by quantifying the binary area of Phalloidin staining, normalized to the mean intensity of DAPI as an indicator of cell number [27].

### Cell viability and proliferation

BeWo cells were incubated with Calcein AM (Thermo Scientific; C1430; 1:200) and Ethidium homodimer-1 (Thermo Scientific; E1169; 1:200) and imaged using an Eclipse Ti-E Inverted Fluorescence Microscope (Nikon). Green fluorescence indicated live cells and red fluorescence indicated dead cells. Images were analyzed using Fiji (National Institutes of Health) and NIS Elements software (Nikon), and the percentage ratio of live to dead cells were calculated by dividing the mean intensity of live cells (as determined by fluorescence of Calcein AM stain) by the mean intensity of the dead cells (as determined by fluorescence of Ethidium homodimer-1 stain).

Cell proliferation was determined using a CellTiter 96® AQueous Non-Radioactive Cell Proliferation Assay kit (MTS Assay; Promega). The absorbance was measured at 490 nm on a Multiskan® Spectrum spectrophotometer (Thermo Scientific). Given that the absorbance is directly proportional to the number of live cells, relative rate of cell proliferation was determined by calculating the fold change in absorbance compared to the 2D control. Matrigel samples with no cells seeded were used to correct for any potential background absorbance.

### RNA extraction and real-time Quantitative Polymerase Chain Reaction (qPCR)

Cells were isolated from hydrogels using Cell Recovery Solution (Corning). Total RNA was extracted from cells using TRIzol Reagent (Invitrogen) and Direct-zol RNA MiniPrep Kit (Zymo Research), following the manufacturer’s protocol. A total of 1 μg of RNA was reverse-transcribed to cDNA using High-Capacity cDNA Reverse Transcription Kit (Applied Biosystems). Primer sets directed against gene targets of interest were designed through National Center for Biotechnology Information’s Primer-BLAST primer designing tool and synthesized at McMaster’s Mobix Labs (**Table 1**). Quantitative analysis of mRNA expression was performed via qPCR using fluorescent nucleic acid dye PerfeCTa SYBR Fastmix (Quanta) and CFX384 Touch Real-Time PCR Detection System (BioRad). The cycling conditions were 95°C for 10 min, followed by 40 cycles of 95°C for 10 secs and 60°C for 10 secs and 72°C for 15 secs. Relative fold changes were calculated using the comparative cycle times (Ct) method, normalizing all values to an endogenous control gene (18S). The endogenous control gene was selected based on experimentally-determined Ct stability across all treatment groups. Given that all primer sets had equal priming efficiency, the ΔCt values for each primer set were calibrated to the average of all control Ct values, and the relative abundance of each primer set compared with calibrator was determined by the formula 2^ΔΔCt^, in which ΔΔCt was the normalized value. Matrigel samples with no cells seeded were also analyzed to ensure that any potential traces of RNA found in hydrogels alone did not confound measurements.

**Table 1:** Forward and reverse sequences for the primers used for qPCR.

Gene	Forward	Reverse	GenBank
*18S (RNA18S5)*	CACGCCAGTACAAGATCCCA	AAGTGACGCAGCCCTCTATG	NR_003286.2
*Glial Cells Missing Homolog 1 (GCM1)*	CCTCTGAAGCTCATCCCTTGC	ATCATGCTCTCCCTTTGACTGG	NM_003643.3
*Placental Lactogen (PL)*	GCCATTGACACCTACCAG	GATTTCTGTTGCGTTTCCTC	V00573.1
*Endogenous Retrovirus Group W Member 1, Envelope; Syncytin-1 (ERVWE1)*	GTTAATGACATCAAAGGCACCC	CCCCATCTCAACAGGAAAACC	NM_014590
*Endogenous Retrovirus Group FRD Member 1, Envelope; Syncytin-2 (ERVFRD1)*	GCCTACCGCCATCCTGATTT	GCTGTCCCTGGTGTTTCAGT	NM_207582.2
*Chorionic Gonadotropin, Alpha (CGA)*	GCAGGATTGCCCAGAATGC	TCTTGGACCTTAGTGGAGTGG	V00518.1
*Chorionic Gonadotropin,* *Beta (CGB)*	ACCCCTTGACCTGTGAT	CTTTATTGTGGGAGGATCGG	J00117.1
*Matrix Metalloproteinase (MMP) 2*	TCTCCTGACATTGACCTTGGC	CAAGGTGCTGGCTGAGTAGATC	NM_004530.5
*MMP9*	CCGGCATTCAGGGAGACGCC	TGGAACCACGACGCCCTTGC	NM_004994.2
*Tissue Inhibitor Of Metalloproteinases (TIMP)1*	GGGCTTCACCAAGACCTACA	TGCAGGGGATGGATAAACAG	NM_003254.2
*TIMP2*	GAAGAGCCTGAACCACAGGT	GGGGGAGGAGATGTAGCAC	NM_003255.4
*Integrin Subunit Alpha (ITGA) 1*	CAGTCTATCCACGGAGAAATG	GGCTCAAAATTCATGGTCAC	NM_181501.1
*ITGA5*	CCAAAAGAAGCCCCCAGCTA	TCCTTGTGTGGCATCTGTCC	NM_002205.4
*ITGAV*	TCACTAAGCGGGATCTTGCC	AGCACTGAGCAACTCCACAA	EF560727.1
*Integrin Subunit Beta (ITGB) 3*	GAAGCAGAGTGTGTCACGGA	TGCATCATTCCTCCAGCCAA	NM_000212.2

### Enzyme-Linked Immunosorbent Assay (ELISA)

Cell media was collected and protein levels of secreted hCGβ were analyzed via ELISA. In brief, 96-well high binding polystyrene microtiter plates (Costar) were coated with a detector anti-hCGβ antibody (Meridian; mAF05-19; monoclonal mouse; 2.94 μg/mL) for 2 hours, and then blocked with 1% BSA in tris-buffered saline overnight at 4°C. Media samples were then incubated in the wells for 2 hours. Plates were incubated with a reporter anti-hCG antibody (Hytest; 27E8; monoclonal mouse; 0.3 μg/mL) conjugated to horseradish peroxidase for 1 hour. Plates were then incubated with TMB substrate (Sigma; T8665) for 30 minutes. Absorbances was measured at 550 nm and 450 nm on a Multiskan® Spectrum spectrophotometer (Thermo Scientific), and 450 nm values were subtracted from 550 nm values to correct for optimal imperfections in microplate. Protein levels were normalized to the absorbance calculated from the MTS proliferation assay, as the absorbance is directly proportional to the number of live cells (method adapted from [15]).

### Statistical analysis

All statistical analyses were performed using Prism 5 software (GraphPad). Results were expressed as means of normalized values ± standard error of the mean (SEM). Experiments were repeated at least three times (n≥3), unless otherwise specified. The significance of differences (p<0.05) between normalized mean values were then evaluated using unpaired t-test or one-way analysis of variance (ANOVA) followed by Tukey’s post-test, as appropriate for the experiment.

## Results

### ECM surface type and thickness differentially regulates cellular organization and morphology

**Fig 1:**
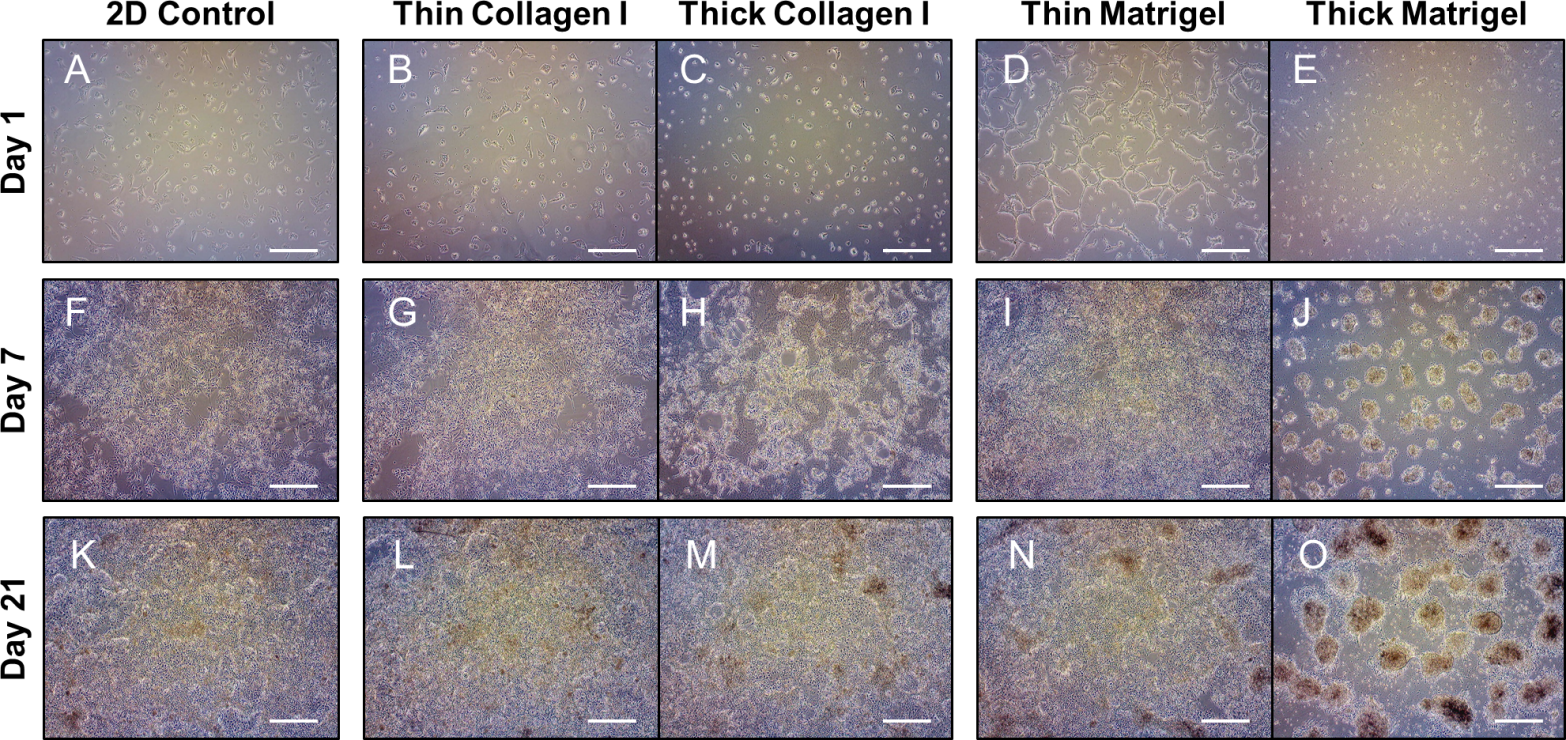
Thick Matrigel regulates self-assembly of trophoblast spheroids as determined by live cell imaging. BeWo cells cultured for 1 day on (A) 2D control surface, (B) thin collagen I, (C) thin Matrigel, (D) thick collagen I, and (E) thick Matrigel. BeWo cells were grown for 7 days on (F) 2D control surface, (G) thin collagen I, (H) thin Matrigel, (I) thick collagen I, and (J) thick Matrigel. BeWo cells were grown for 21 days on (K) 2D control surface, (L) thin collagen I, (M) thin Matrigel, (N) thick collagen I, and (O) thick Matrigel. All images were taken at 4x objective magnification and scale bar indicates 500 μm. n = 3.

BeWo cells were seeded onto 2D polystyrene surfaces, or thin or thick surfaces of collagen I or Matrigel, and live cellular organization was examined using phase-contrast microscopy. Differences in cellular organization were seen within 24 hrs of seeding (day 1; **Fig 1A–1E**), with long, strand-like morphologies particularly evident on thin Matrigel surfaces (**Fig 1D**), and small aggregates seen on thick surfaces (**Fig 1E**). By day 7, BeWo cells cultured on thin collagen I and Matrigel appeared more densely populated than the 2D control, but retained sheet-like, confluent growth and were no longer distinct in terms of cellular organization (**Fig 1F, 1G and 1I**). However, BeWo cells cultured on thick collagen I formed undefined aggregates at day 7 (**Fig 1H**), whereas cells cultured on thick Matrigel self-assembled into distinct, spheroid-shaped aggregates at day 7 (**Fig 1J**). By day 21, BeWo cells cultured on thin surfaces no longer appeared different in organization compared to the 2D control (**Fig 1K, 1L and 1N**). In contrast, the cultures grown on thick collagen I samples did not exhibit differences compared to the 2D control (**Fig 1K and 1M**). Notably, thick Matrigel-induced trophoblast spheroids maintained in shape and integrity, but grew in size from day 7 to day 21 (**Fig 1J and 1O**). Collectively, thick surfaces were required for aggregate formation, and thick Matrigel was specifically required for spheroid self-assembly and maintenance.

### Matrigel led to thickness-dependent increases in cell viability and proliferation

**Fig 2:**
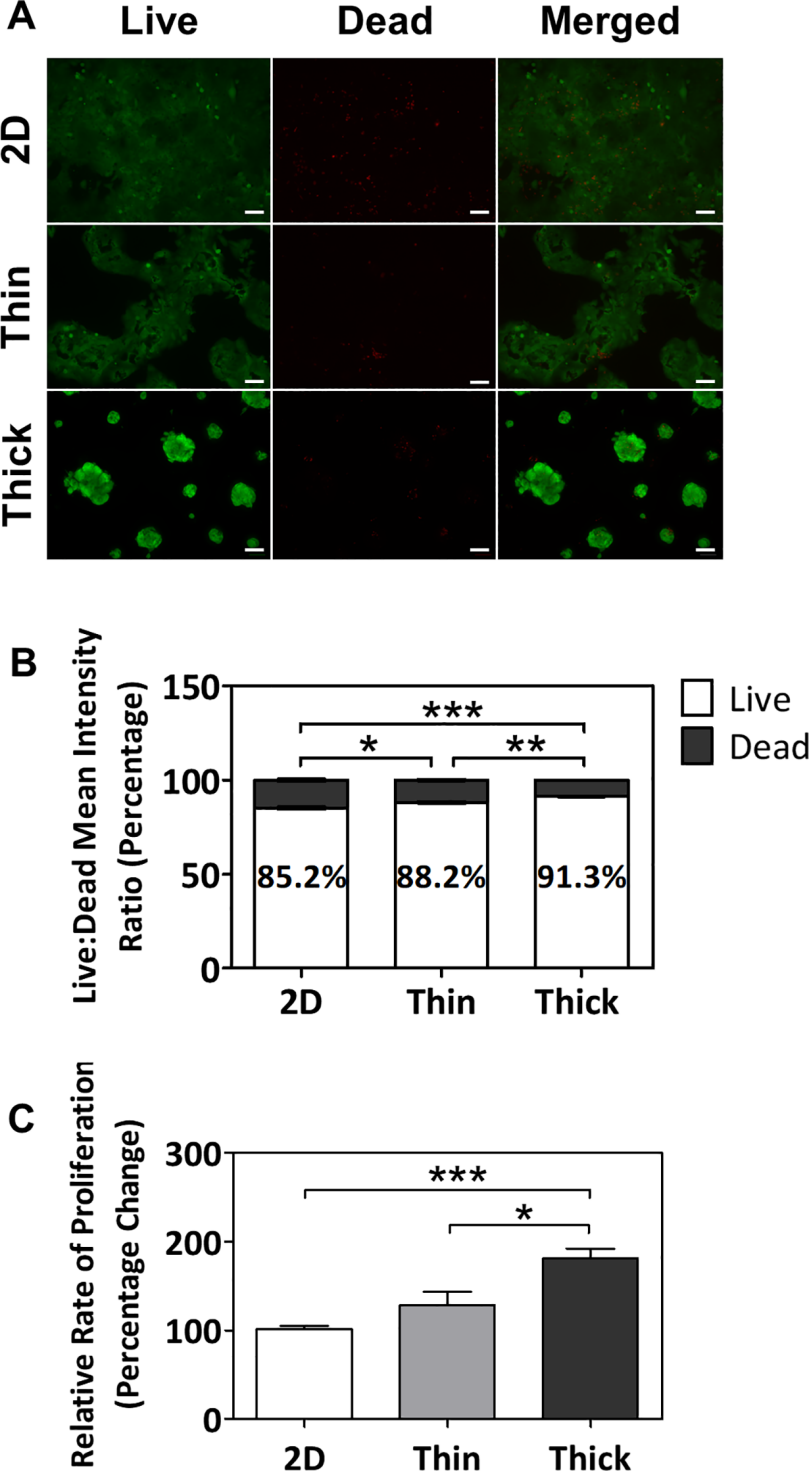
Thickness-dependent increases in cell viability and proliferation in BeWo cells cultured on Matrigel after 7 days. (A) Immunofluorescent images of BeWo cells stained with Calcein AM (green) and Ethidium homodimer-1 (red). All images were taken at 10x objective magnification and scale bar indicates 100 μm. (B) Percentage ratio of mean intensities of live and dead cells cultured on 2D, thin, and thick surfaces. (C) Relative rates of proliferation of cells cultured on 2D, thin, and thick surfaces as assessed via MTS assay. Significant differences between treatment groups determined by one-way ANOVA followed by Tukey’s post-test; n ≥ 3. Significant differences between means determined by post-tests were indicated by * (p<0.05), ** (p<0.01), or *** (p<0.001).

Self-assembling, 3D cell spheroids and microtissues are of great interest as they have been shown to better recapitulate the phenotypes redolent of their respective organs compared to two-dimensional (2D) cultures, such as tissue-specific cell density, microarchitecture, cell-cell interactions [10, 28–31]. Given the potential value, we further characterized the thick Matrigel-induced trophoblast spheroids at day 7. Cell viability, as determined through the ratio of live to dead cells, significantly increased in a thickness-dependent manner (p<0.05 for 2D to thin; p<0.001 for 2D to thick; p<0.01 for thin to thick; **Fig 2A and 2B**), with the greatest viability in cells grown on the thick Matrigel surface (91.3 ± 0.2%). Interestingly, the mean of the cell proliferation rate appeared to increase in a thickness-dependent manner, with significant differences evident in the thick Matrigel surface when compared to the other two groups (181.0 ± 29.3%, p<0.001 for 2D to thick; p<0.05 for thin to thick; **Fig 2C**).

### The effect of ECM surface thickness on syncytial fusion

**Fig 3:**
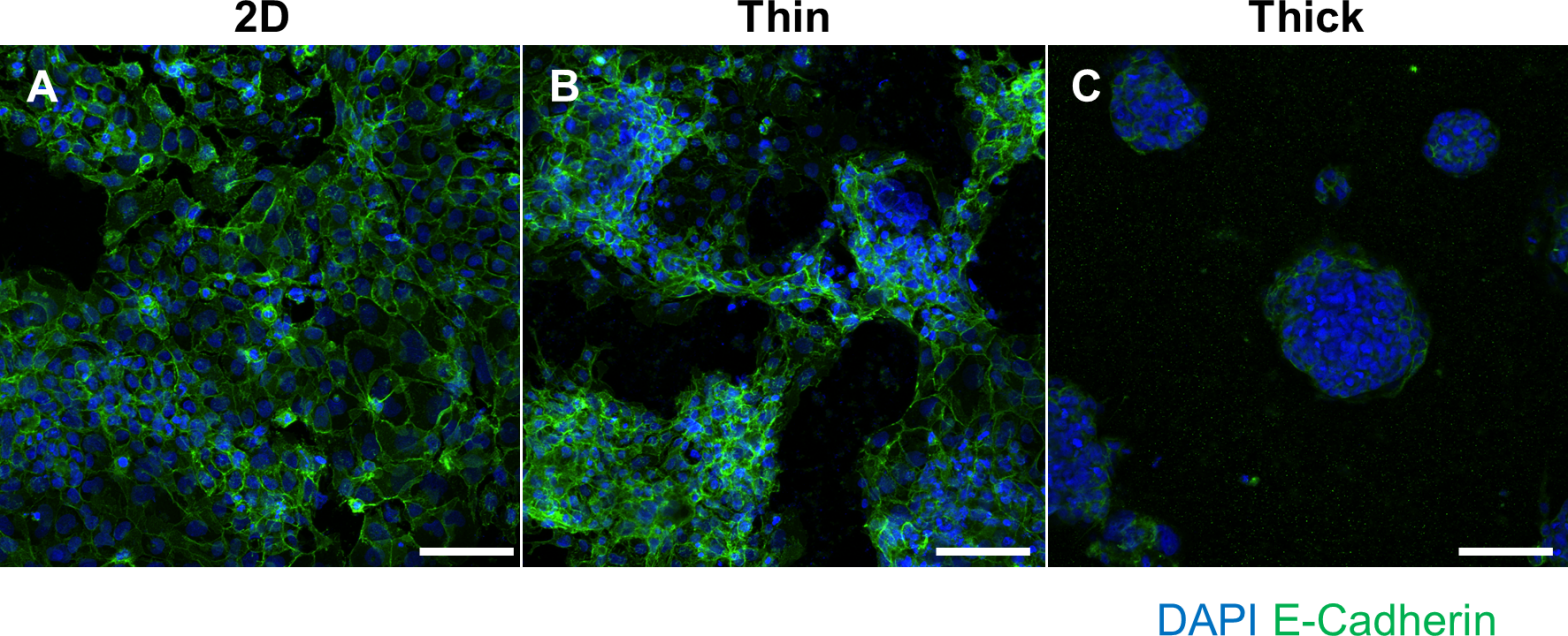
Immunofluorescent staining of E-Cadherin and DAPI to visualize syncytial fusion. BeWo cells grown on (A) 2D, (B) thin Matrigel, or (C) thick Matrigel surfaces. Green fluorescence indicates E-Cadherin staining and blue fluorescence indicates DAPI staining for cell nuclei. Images were taken at 20x magnification and scale bar indicates 100 μm.

Due to the robust effects on cellular organization, spheroid self-assembly, viability, and proliferation, we investigated the impact of surface thickness on syncytial fusion, which is an essential feature of syncytiotrophoblast differentiation. Interestingly, BeWo cells grown on thick Matrigel appeared to be very highly fused with minimal E-Cadherin staining at the center of spheroids compared to cells grown on 2D or thin Matrigel (**Fig 3A–3C**). However, due to the high density of DAPI-positive nuclear clustering in thin and thick Matrigel surfaces, it was not possible to accurately distinguish nuclei from one another, preventing the accurate quantification of the percentage of syncytial fusion. Thus, we assessed the degree of syncytial fusion through gene expression profiling to verify this qualitative fusion increase seen in thick Matrigel-induced spheroids.

**Fig 4:**
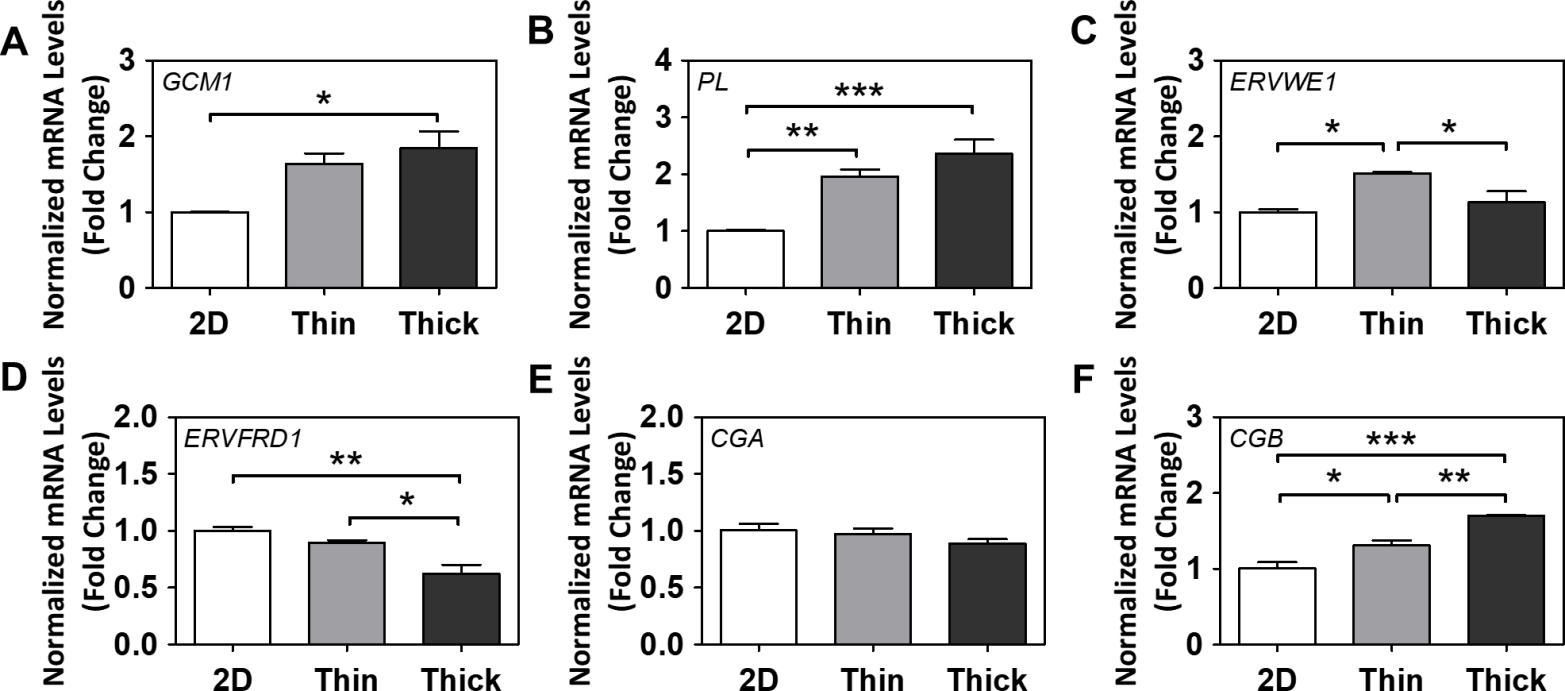
The effect of Matrigel thickness on gene markers of differentiation and syncytial fusion. Normalized mRNA levels of (A) *GCM1*, (B) *PL*, (C) *ERVWE1*, (D) *ERVFRD1*, (E) *CGA*, and (F) *CGB* after 7 days of growth on various surface thicknesses. (E) Normalized protein levels of secreted hCGβ in media. Significant differences between treatment groups determined by one-way ANOVA followed by Tukey’s post-test; n≥3. Significant differences between means determined by post-tests were indicated by * (p<0.05), ** (p<0.01), or *** (p<0.001).

Thick Matrigel-induced spheroids exhibited a significant two-fold increase in mRNA levels of glial cells missing homolog 1 (*GCM1*), a transcription factor for many syncytialization-related genes [32], compared to cells grown on 2D surfaces (p<0.05; **Fig 4A**). Placental lactogen (*PL*) mRNA levels were significantly increased in a thickness-dependent manner (p<0.01 for 2D to thin; p<0.001 for 2D to thick; **Fig 4B**). Endogenous retrovirus group W member 1 (*ERVWE1*) mRNA levels only increased in the thin Matrigel group (p<0.05 for 2D to thin; **Fig 4C**), but endogenous retrovirus group FRD member 1 (*ERVFRD1*) mRNA levels contrastingly decreased (p<0.01 for 2D to thick; p<0.05 for thin to thick; **Fig 4D**). Human chorionic gonadotropin α (*CGA*) mRNA levels were unchanged (**Fig 4E**), but human chorionic gonadotropin β (*CGB*) mRNA levels were significantly increased in a thickness-dependent manner (p<0.05 for 2D to thin; p<0.001 for 2D to thick; p<0.01 for thin to thick; **Fig 4F**). Collectively, surface thickness/stiffness alone was able to induce increases in several key markers of syncytial fusion (*GCM1*, *PL*, *ERVWE1*, *CGB*).

**Fig 5:**
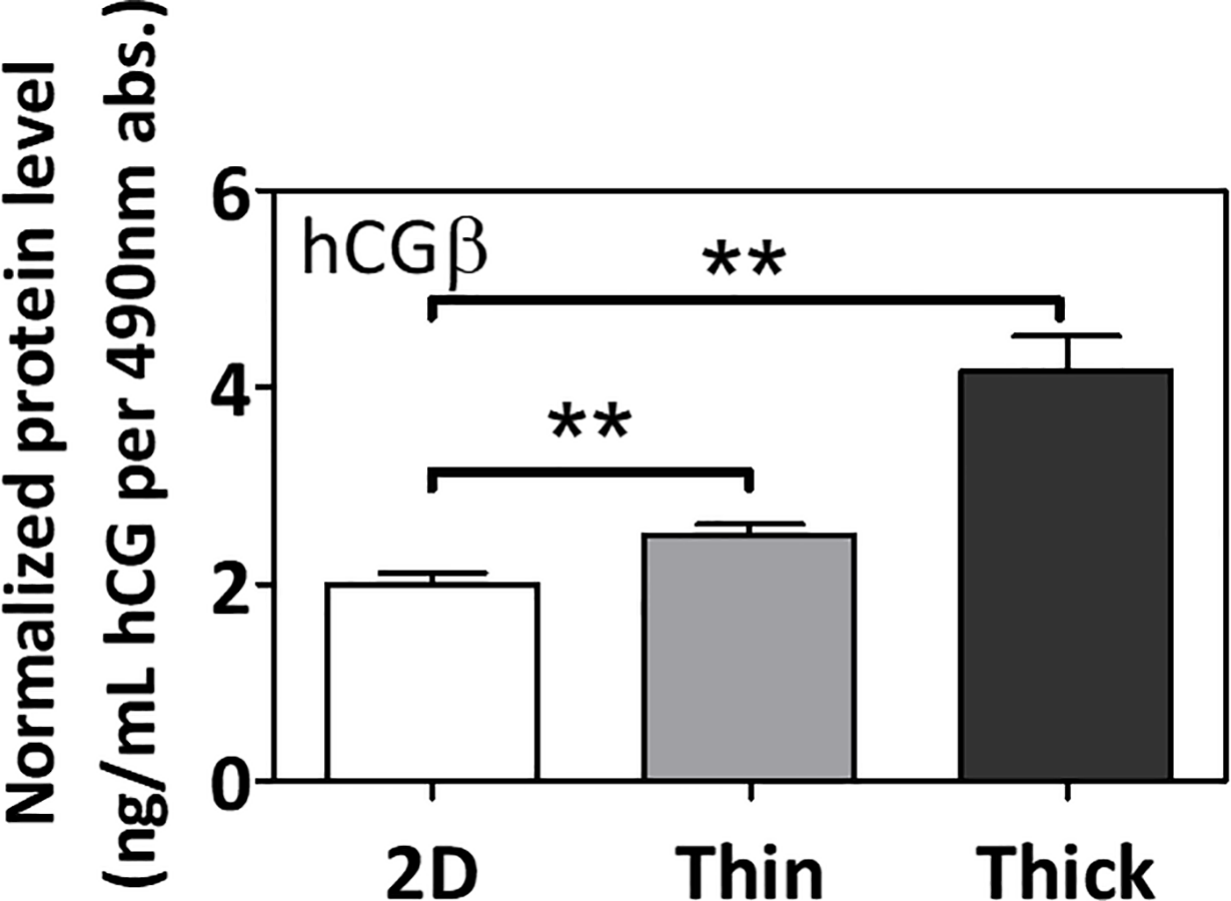
The effect of Matrigel thickness on the secretion of human chorionic gonadotropin β (*hCGβ)* in the cell media. Normalized protein levels of secreted hCGβ in media. Significant differences between treatment groups determined by one-way ANOVA followed by Tukey’s post-test; n≥3. Significant differences between means determined by post-tests were indicated by ** (p<0.01).

In accordance, surface thickness alone also induced significant increases in secreted protein levels of human chorionic gonadotropin β (*hCGβ)* in the cell media (p<0.05; **Fig 5**).

### Cellular stiffness response to changes in ECM surface thickness

**Fig 6:**
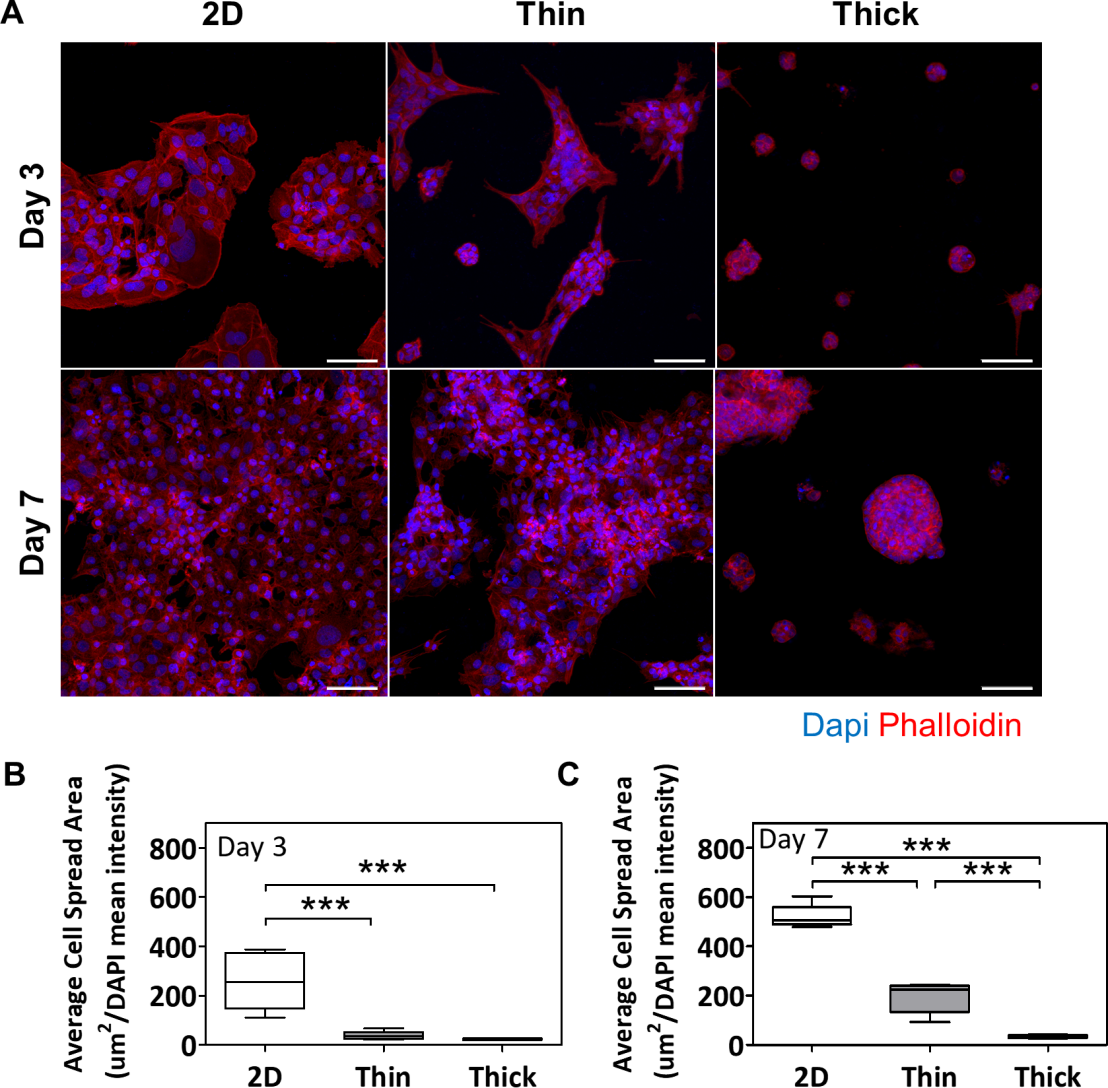
Thick Matrigel leads to decreased F-actin cell spread areas. (A) Immunofluorescent images of Phalloidin staining at days 3 and 7 across various surface thicknesses. Red fluorescence indicates phalloidin staining for F-actin and blue fluorescence indicates DAPI staining for cell nuclei. Images were taken at 20x magnification and scale bar indicates 100 μm. Average cell spread areas as determined by quantifying the normalized binary area of phalloidin stain at (B) day 3 and (C) day 7. Significant differences between treatment groups determined by one-way ANOVA followed by Tukey’s post-test; n = 3. Significant differences between means determined by post-tests were indicated by *** (p<0.001).

As substrate stiffness inversely correlated with changes in surface thickness, as seen in our findings (**S1 Fig**) and that of others [16], we were interested in further elucidating potential stiffness-sensing mechanisms involved in spheroid formation with the thick Matrigel ECM. The cells’ ability to spread over a surface is a known stiffness-response marker [16], and may be assessed via F-actin (phalloidin) immunofluorescent staining. At day 3, cell spread areas were significantly decreased as surface thickness increased (p<0.001; **Fig 6A and 6B**), and a similar trend was present at day 7 (p<0.001; **Fig 6A and 6C**).

### Thick ECM surface up-regulated genes related to stiffness sensing and invasion

**Fig 7:**
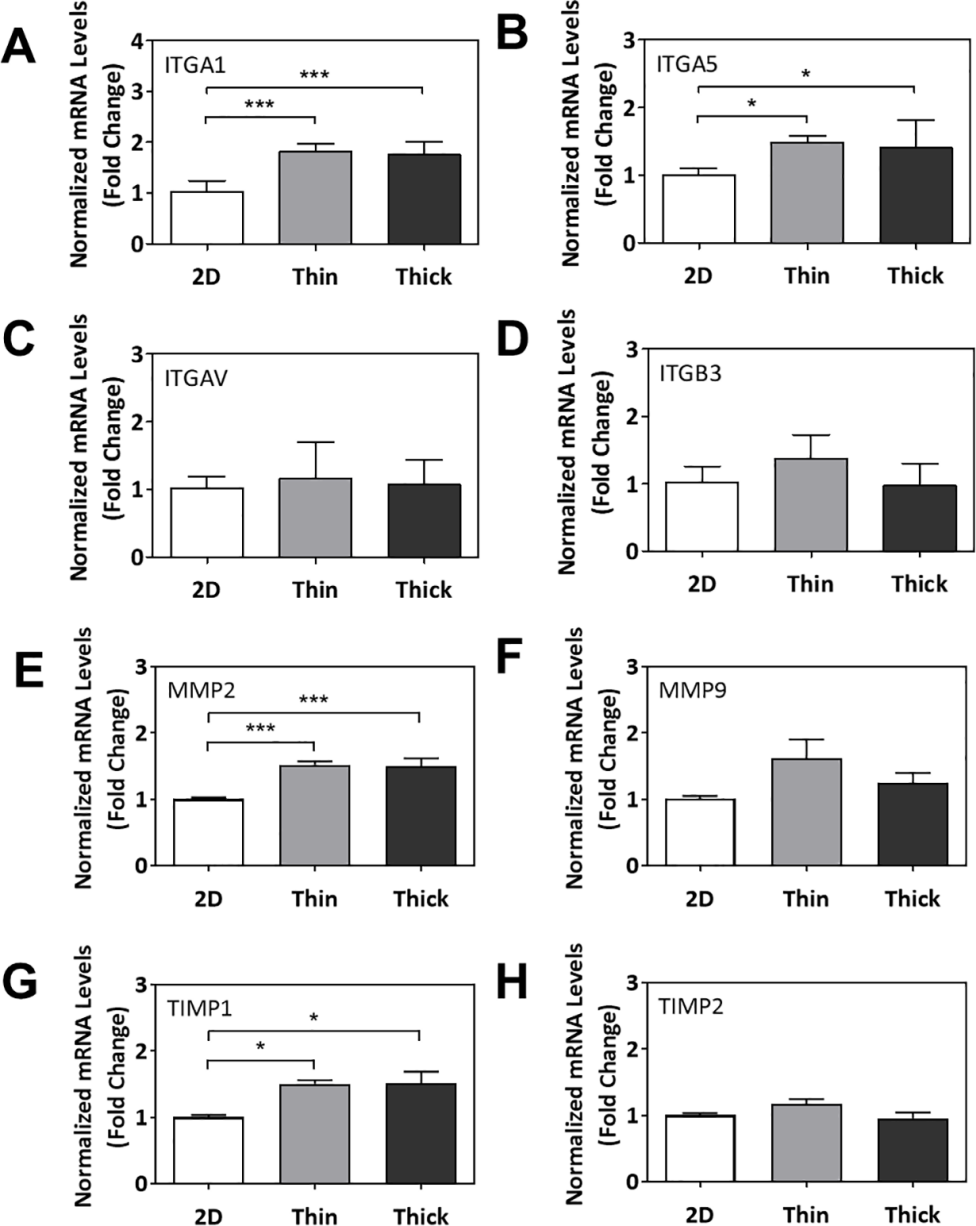
Gene expression profiling of cellular stiffness response to ECM surface thickness. Normalized mRNA levels of (A) *ITGA1*, (B) *ITGA5*, (C) *ITGAV*, (D) *ITGB3*, (E) *MMP2*, (F) *MMP9*, (G) *TIMP1*, and (H) *TIMP2*. Significant differences between treatment groups determined by one-way ANOVA followed by Tukey’s post-test; n≥3. Significant differences between means determined by post-tests were indicated by * (p<0.05), ** (p<0.01), or *** (p<0.001).

Lastly, we investigated the impact of Matrigel thickness on expression of genes related to stiffness sensing and invasion/migration. At day 7, *ITGA1* and *ITGA5* mRNA levels significantly increased in cells grown on thin and thick Matrigel surfaces compared to the 2D control (p<0.001 and p<0.05, respectively; **Fig 7A and 7B**). *MMP2* and *TIMP1* mRNA levels also significantly increased in cells grown on thin and thick Matrigel surfaces compared to the 2D control (p<0.001 and p<0.05, respectively; **Fig 7E and 7G**). mRNA levels of *ITGAV*, *ITGB3*, *MMP9*, *and TIMP2* did not significantly change across various surface thicknesses (**Fig 7C, 7D, 7F and 7H**).

## Discussion

The current study demonstrates that the nature of the ECM alone impacts not only the self-assembly behaviour of trophoblast cells, but also the expression profiles of genes related to differentiation and cell-ECM interaction, and functionally alter syncytial fusion and hormone secretion. The ability to manipulate surface thickness, as a parameter to alter substrate stiffness, allows for the evaluation of how cellular function and phenotype are regulated by changing ECM stiffness without altering the composition of the ECM hydrogel. The importance of exploring such relationships is underscored in reports that have demonstrated that self-assembling spheroids are of great value as they are known to possess cellular interactions and densities that are more similar to the *in vivo* state than 2D cultures [10, 29, 30]. While the generation of placental trophoblast spheroids has been attempted by a few prior studies [33–36], the cellular phenotype and techniques required for their derivation had yet to be well-characterized. Some studies utilized non-adherent or rotating wall vessel bioreactors to generate spheroids, but these models lack the cell-ECM interactions that are essential *in vivo* [33–36]. Our study reveals the importance of understanding the cell-ECM interactions to influence cell-cell interactions, as seen through spheroid formation. Importantly, the novelty in our work demonstrates that the physical properties of the ECM contributes to not only to cellular reorganization, but also alters key cellular functions such as the trophoblasts’ secretion of hCGβ, which is crucial in regulating hormone production, trophoblast fusion and invasion, and many other aspects of maternal and fetal health *in vivo* [37, 38]. This connection between ECM and cellular function may prove to be a vital factor in dictating adverse pregnancy outcomes. Therefore, *in vitro* models of placental function using trophoblasts should consider clearly defining the ECM used within these constructs.

In the current manuscript, we demonstrate that the type of ECM plays a key role in regulating the self-assembly and maintenance of 3D trophoblast spheroids in BeWo cells. The differential abilities of collagen I and Matrigel in maintaining spheroid integrity is consistent with the work of Nguyen-Ngoc, Cheung (39) showing that human breast cancer cells exhibited greater disassociation from pre-formed tumour spheroids when grown on or embedded in collagen I compared to Matrigel alone [19, 39]. Collectively, this suggests that Matrigel is a more appropriate biomaterial than collagen I at maintaining 3D trophoblast spheroid integrity. Furthermore, the additional ECM proteins present in Matrigel (*e*.*g*., laminin, entactin) compared to 2D surfaces or collagen I alone may also contribute to cellular differentiation. For example, knocking out laminin (α5 subunit) led to placental abnormalities and embryonic lethality in mice [40], and silencing laminin α4 or its receptor led to impaired trophoblastic functions (*e*.*g*., decreased invasion, migration, and tube formation) in human placental trophoblast cells, suggesting unique roles among the varying ECM proteins in development and differentiation. Indeed, our thick Matrigel-driven trophoblast spheroids exhibited higher degrees of syncytial fusion and gene (*GCM1*, *PL*, *ERVWE1*, *CGB*) and protein (hCGβ) expression profiles indicative of a more differentiated population compared to cells grown on 2D surfaces. The lack of change seen in *CGA* mRNA levels are not particularly surprising given the known differential temporal regulation of *CGA* and *CGB* expression during pregnancy (where hCGβ normally peaks around 10–12 weeks, whereas hCGα increases gradually until term. Importantly, the extent of fusion evident in the BeWo cells cultured on thick Matrigel were visibly greater than on 2D surfaces in conjunction with increases in hCGβ secretion, collectively supporting the enhancement of cell fusion via increased ECM thickness and/or decreased stiffness. In addition to activating cellular differentiation, ECM surfaces were previously reported to activate cellular invasion [11, 33, 34]. During invasion, MMP expression and activity is increased to degrade collagens [41–44], which was also seen in our study. A hydrogel surface solely consisting of isolated collagen, such as collagen I, is likely to be more impacted by the degradation compared to an ECM protein cocktail-based hydrogel, like Matrigel, when faced with invasive, MMP-expressing trophoblasts [41–44]. Therefore, the lack of spheroid structures on collagen I at day 21 may be attributed to higher degrees of surface degradation over time, enabling cells to contact the coverslip and return to confluent growth, whereas Matrigel remained more robust as a scaffold due to a more diverse ECM composition. However, supplementary studies characterizing the ECM protein composition, and actual changes to integrity and surface topography of these surfaces during and after trophoblast invasion are required to verify these speculations.

While hydrogel surface-induced placental cell aggregation was previously reported by a small number of other studies [11–13, 15], our study provides a more precise definition of the surface-casting parameters (*e*.*g*., surface thickness and stiffness). Our data suggests that a critical surface thickness is required for spheroid formation, and variations in thickness can regulate spheroid phenotype. Kliman and Feinberg (14) cultured primary trophoblasts and JEG3 cells on a gradual slope of Matrigel (thicknesses reported between 0–60 μm), and elegantly demonstrated variations in cell morphology across the thicknesses [14]. Although they did not report spheroid formation due to the short duration of their study (24–72 hours), they did see rounded and individually-seeded cells when cultured on 14–60 μm-thick Matrigel, which resembled a pre-spheroid state. Interestingly, their placental cells eventually entirely degraded thinner coats of Matrigel to resume growth on the underlying glass coverslip [14]. This provides a plausible explanation for why the distinct strand-like cellular formations seen in BeWo cells cultured on thin Matrigel at day 1 were not maintained over time. The thickness-dependent changes seen throughout our study also propose that the cells can grade their behaviour based on sensing the actual thickness of the surface, or perhaps by sensing another property directly affected by thickness, such as stiffness. Indeed, crosslinking poly(ethylene) glycol networks within Matrigel to increase gel stiffness was demonstrated to alter the invasion and dispersion behaviours of mammary organoids and mesenchymal stem cells [17, 45]. Others have also consistently reported increased invasive activity on ECM surfaces *in vitro* in various cell types [39, 46–48]. We tested this hypothesis in our model through mechanical testing to show that ECM thickness was inversely correlated with stiffness. Moreover, the quantitative reduction in cell spreading correlates with reduction in matrix stiffness and suggests that trophoblast cells possess the ability to sense the ECM thickness via stiffness-sensing and invasion mechanisms. This was similarly seen in the work of Mullen *et al*. (2015) in osteogenic cells [16]. Interestingly, even in the absence of a traditional stimulus for invasion (*i*.*e*., nutrient or oxygen gradient), small, but significant, increases in *ITGA1*, *ITGA5*, *MMP2*, and *TIMP1* mRNA levels were detected in BeWo cells cultured on both thin and thick Matrigel, likewise demonstrating that the presence of ECM is sufficient to induce expression of some underlying genes. It is understood that the integrin subunits αv, α5, α1 and/or β3 link to the actin cytoskeleton through focal adhesion kinase anchoring points, regulating MMP expression and subsequent cellular invasion via a cellular mechano-sensing pathway [49–53]. However, though capable of invasion, BeWo cells traditionally display a less invasive phenotype [54], which may explain in part why we did not observe robust changes across all the genes (*e*.*g*., *ITGAV*, *ITGB3*, *MMP9*, *and TIMP2*). Additional studies should investigate the effects of a stronger stimulus for invasion (*i*.*e*., nutrient or oxygen gradient) on actual spheroid invasion and whether additional invasion/migration genes (*ITGAV*, *ITGB3*, *MMP9*, *TIMP2*, *etc*.) may also change.

Spheroid formation also coincided with thickness-dependent increases in expression of several syncytialization-related genes, *GCM1*, *PL*, *CGB*, and its secreted protein product, hCGβ. While human CG is produced by several placental cell types, its main source is from syncytiotrophoblasts–the fusogenic, non-proliferative, terminally-differentiated, endocrine cells [55]. In effect, increased CG expression in our trophoblast spheroids acts as a biochemical marker of syncytiotrophoblast differentiation and fusion [32, 56], thereby, providing semi-quantitative support for the increased syncytial fusion seen in immunofluorescent images of thick Matrigel-derived spheroids. Interestingly, these increases coincided with minimally-changed *ERVWE1* and decreased *ERVFRD1* mRNA levels. While the syncytins have been demonstrated to play a role in syncytial fusion, the timing of their expression and functional involvement remain unclear [57]. For example, *Syncytin-B* knock-out mice (analogous to *ERVFRD1/*syncytin-2 in humans) resulted in abnormal placentation, but the placentas still exhibited some syncytialization and the offspring were viable, suggesting compensatory mechanisms or the existence of alternative fusogenic proteins [58]. Taken together, our data suggest that the Matrigel ECM potentiates modest syncytial fusion, even in the absence of fusogenic agents such as forskolin, but future studies are required to further profile and characterize the intricate gene expression patterns underlying these changes. This increased differentiation and syncytialization achieved further validates the importance of the ECM conditions in modelling placentation and emphasizes the benefits of spheroid formation in trophoblast cultures.

## Conclusion

As the sophistication of *in vitro* research grows through the incorporation of ECM biomaterials, so does the necessity to better characterize the biological response of cells involved. Bearing in mind the collective implications on cellular organization, behaviour, and differentiation-related gene and protein expression profiles, our findings emphasize the importance of characterizing the ECM surface parameters used in spheroid/organoid-based assays and cultures. The generation of self-assembling spheroid cultures through regulation of ECM surface type and thickness also contributes to a deeper understanding of cell-ECM interactions. In consideration of the increased usage of 3D bioprinting and microfluidic “placenta-on-a-chip” devices within the last several years [5, 59–61], a proper understanding and integration of ECM biomaterials will be a crucial step towards generating more *in vivo*-like models.

## Supporting information

S1 Fig Analysis of ECM surface properties. (A) Schematic representing thin and thick ECM surface samples. (B) Representative images of ECM surfaces as captured by MicroSquisher camera. (C) Measurements of actual thicknesses of ECM surface based on theoretical calculations for 50 and 250 μm. (D) Measurements of ECM surface stiffness based on surface thickness. Significant differences between means indicated by *** (p<0.001), as determined by unpaired t-Test; n = 3.
(TIF)
