# Supplementary figures and images for: Extracellular matrix surface regulates self-assembly of three-dimensional placental trophoblast spheroids
Source: PLoS One. 2018 Jun 25;13(6):e0199632. doi: 10.1371/journal.pone.0199632 (PMC6016924; doi:10.1371/journal.pone.0199632)

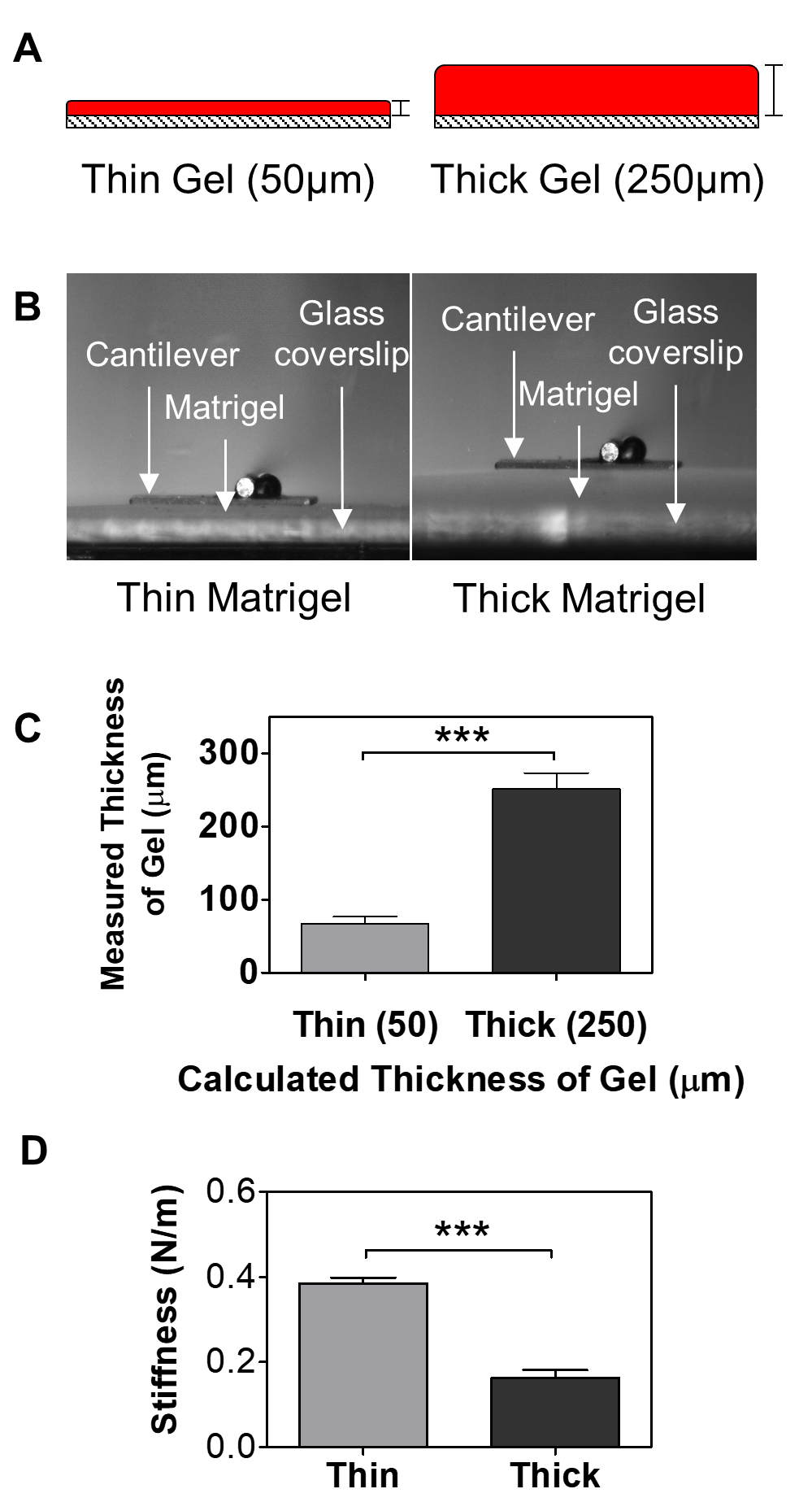

Supplement: S1 Fig — (A) Schematic representing thin and thick ECM surface samples. (B) Representative images of ECM surfaces as captured by MicroSquisher camera. (C) Measurements of actual thicknesses of ECM surface based on theoretical calculations for 50 and 250 μm. (D) Measurements of ECM surface stiffness based on surface thickness. Significant differences between means indicated by *** (p<0.001), as determined by unpaired t-Test; n = 3. (TIF) [file pone.0199632.s001.TIF]
